# Diabetes, plasma glucose and incidence of colorectal cancer in Chinese adults: a prospective study of 0.5 million people

**DOI:** 10.1136/jech-2018-210651

**Published:** 2018-07-03

**Authors:** Yuanjie Pang, Christiana Kartsonaki, Yu Guo, Yiping Chen, Ling Yang, Zheng Bian, Fiona Bragg, Iona Y Millwood, Leijia Shen, Songgen Zhou, Jiben Liu, Junshi Chen, Liming Li, Michael V Holmes, Zhengming Chen

**Affiliations:** 1 Clinical Trial Service Unit & Epidemiological Studies Unit (CTSU), Nuffield Department of Population Health, University of Oxford, Oxford, UK; 2 Medical Research Council Population Health Research Unit (MRC PHRU), Nuffield Department of Population Health, University of Oxford, Oxford, UK; 3 Chinese Academy of Medical Sciences, Beijing, China; 4 Tongxiang Renmin Hospital, Tongxiang, China; 5 Wuzhen Central Hospital, Tongxiang, China; 6 Yongqing Road Community Health Center, Qingdao, China; 7 National Center for Food Safety Risk Assessment, Beijing, China; 8 School of Public Health, Peking University, Beijing, China; 9 National Institute for Health Research Oxford Biomedical Research Center, Oxford University Hospital, Oxford, UK

**Keywords:** diabetes, blood glucose, colorectal cancer, Chinese

## Abstract

**Background:**

Diabetes is associated with higher risk of colorectal cancer (CRC). Uncertainty remains about the relevance of duration of diabetes and about the association of blood glucose with CRC risk among individuals without diabetes.

**Methods:**

The prospective China Kadoorie Biobank recruited 512 713 participants in 2004–2008 from 10 diverse areas in China. After 10 years of follow-up, 3024 incident cases of CRC (1745 colon, 1716 rectal) were recorded among 510 136 participants without prior cancer at baseline. Cox regression was used to estimate adjusted HRs for CRC associated with diabetes (previously diagnosed or screen-detected) and, among those without previously diagnosed diabetes, with levels of random plasma glucose (RPG).

**Results:**

Overall 5.8% of participants had diabetes at baseline. Individuals with diabetes had an adjusted HR of 1.18 (95% CI 1.04 to 1.33) for CRC, with similar risk for colon and rectal cancer (1.19 [1.01 to 1.39] vs 1.14 [0.96 to 1.35]). The HRs decreased with longer duration of diabetes (*p* for trend 0.03). Among those without previously diagnosed diabetes, RPG was positively associated with CRC, with adjusted HRs per 1 mmol/L higher baseline RPG of 1.04 (1.02 to 1.05) for CRC, again similar for colon and rectal cancer (1.03 [1.01to 1.05] and 1.04 [1.02 to 1.06], respectively). The associations of diabetes and RPG appeared stronger in men than in women, but the differences were non-significant (*p* for heterogeneity 0.3 and 0.2).

**Discussion:**

Among Chinese adults, diabetes and higher blood glucose levels among those without known diabetes are associated with higher risk of CRC.

## Introduction

Colorectal cancer (CRC) ranks as the third most common cancer and the fourth leading cause of cancer-related death globally.^1^ The incidence has been rising in low-income and middle-income countries since the mid-1970s including China,^2^ where the proportion of rectal cancer compared with colon cancer is higher than that in most Western populations.^3^ Previous studies of mostly Western populations have shown that individuals with diabetes had an increased risk of CRC,^4^ although the associations by sex and by anatomical site and the relevance of duration of diabetes are less clear. Assessment of duration of diabetes in relation to CRC risk may provide valuable insights into disease aetiology. Among individuals without diabetes, substantial uncertainty also remains as to whether blood glucose levels are associated with risk of CRC and, if so, whether there is any threshold below which the association no longer exists.^5 6^

In China, the prevalence of diabetes has increased from 2.5% in the 1990s to 11.6% in 2013, with approximately 60% of diabetes undetected.^7^ Furthermore, the increase in diabetes prevalence is so recent that its impact on risks of cancer, if any, may not yet have fully emerged. Despite this, there is little reliable prospective evidence available in China about the associations of diabetes and blood glucose, both qualitatively and quantitatively, with risks of CRC.^8^ We examined the associations of diabetes and of blood glucose among participants without previously diagnosed diabetes with risk of CRC within the China Kadoorie Biobank (CKB) population of 0.5 million adults, both overall and by sex and anatomical site.

## Methods

### Study population

Details of the CKB design, survey methods and population characteristics have been described elsewhere.^9^ Briefly, 512 713 participants aged 30–79 years were recruited into the study from 10 geographically defined localities (5 urban and 5 rural) in China during 2004–2008. The study areas were selected to provide diversity in risk exposure and disease patterns, while taking into account population stability, quality of mortality and morbidity registries, capacity and long-term commitment within the areas. Central ethical approvals were obtained from Oxford University and the China National Center for Disease Control and Prevention (CDC). Approvals were also obtained from institutional research boards at the local CDCs in the 10 areas.

### Data collection

At local study assessment clinics, participants completed an interviewer-administered laptop-based questionnaire on sociodemographic characteristics, smoking, alcohol consumption, diet, physical activity, personal and family medical history and current medication. A range of physical measurements were recorded by trained technicians, including height, weight, hip and waist circumference, bio-impedance, lung function, blood pressure and heart rate, using calibrated instruments with standard protocols.

A 10 mL non-fasting (with the time since the participant last ate recorded) blood sample was collected from participants into an EDTA vacutainer (BD Hemogard, BD, USA). Immediate on-site testing of random plasma glucose (RPG) level was undertaken using the SureStep Plus System (Johnson & Johnson), regularly calibrated with manufacturer quality control solution. Participants with RPG levels≥7.8 mmol/L and <11.1 mmol/L were invited to return for a fasting plasma glucose (FPG) test the next day. RPG data were unavailable for 8341 participants (because of a delay in making the on-site test available in certain regions).

Previously diagnosed diabetes was defined by the question ‘Has a doctor ever told you that you had diabetes?’. Among positive respondents, additional information about age at diagnosis and current use of certain medications for the treatment of diabetes (eg, insulin and metformin) and cardiovascular diseases (eg, aspirin, lipid and blood pressure lowering agents) was collected. Among those without previously diagnosed diabetes, screen-detected diabetes was defined as: (1) RPG≥7.0 mmol/L if the time since last eating was ≥8 hour; (2) ≥11.1 mmol/L if the time since last eating was <8 hour or (3) a FPG≥7.0 mmol/L on subsequent testing.^10^ Of all 504 372 participants who provided blood samples, 108 200 participants had fasted for at least 8 hours. Of 26 444 participants who had not fasted and had an elevated RPG, 13 009 participants returned for a FPG test. For screen-detected diabetes, 4413 cases were identified based on RPG among individuals who fasted for ≥8 hours, 8122 cases on RPG among individuals who fasted for <8 hours and 1582 cases on FPG on repeat testing.

From August to October 2008 (~2.6 years after the baseline survey) 19 788 (~5%) surviving participants were randomly selected to attend a resurvey. The data collection and survey procedures were much the same as in the baseline survey. RPG data were available for 19 712 (99.6%) resurvey participants.

### Follow-up for morbidity and mortality

The vital status of each participant was determined periodically through China CDC’s Disease Surveillance Points (DSP) system and national health insurance system, supplemented by regular checks against local residential and administrative records and by annual active confirmation through street committees or village administrators.^11^ In addition, information about death, major diseases and any episodes of hospitalisation was collected through linkage, via each participant’s unique national identification number, with death registries, disease registries (for cancer, ischaemic heart disease, stroke and diabetes) and national health insurance claims databases. All death or hospitalised disease events were coded using International Classification of Diseases, 10th Revision (ICD-10) by trained DSP staff who were blinded to baseline information, and reviewed centrally for consistency. Information on cancer histological subtypes was also collected for a subset of the cases through cancer registries or reviews of hospital medical notes as part of the ongoing outcome adjudication for major diseases. By 1.1.2016, 41 410 (8%) participants had died, 5201 (1.0%) were lost to follow-up and 26 595 (5%) had developed cancer. Distribution and classification of CRC and are shown in online supplementary table 1.

10.1136/jech-2018-210651.supp1 Supplementary file 1

### Statistical analysis

We excluded individuals with a prior history of cancer at baseline (n=2577), leaving 510 136 individuals for the main analysis (online supplementary figure 1). Mean values and prevalence of baseline characteristics and incidence rates of CRC were calculated by body mass index (BMI) categories, using direct standardisation to the age (5 year groups), sex and area structure of the population, where appropriate.

Cox proportional hazards models were used to estimate adjusted HRs of CRC incidence associated with diabetes and RPG levels at baseline, stratified by age-at-risk (5 year groups), sex and study area (10 areas) and adjusted for age at baseline, education (six groups: no formal school, primary school, middle school, high school, technical school/college or university), smoking (three groups: never regular, occasional, former regular or current regular), alcohol (five groups: abstainers, ex-weekly drinkers, reduced-intake drinkers, occasional drinkers or weekly drinkers) and total physical activity. Cox models with a time-updated exposure for diabetes duration were used to estimate the association of diabetes duration with CRC risk, with the same adjustment. Incident diabetes cases (ICD-10 code E11, n=16 431) that occurred during the follow-up were also included. We also used Cox models with a time-updated exposure in which individuals with incident diabetes were considered as exposed from their time of diagnosis. Duration was defined as the time interval between diabetes diagnosis and time at risk. The analysis for RPG was conducted in participants without previously diagnosed diabetes (n=486 189). RPG was categorised into five groups (≤5.5 [reference], 5.6–6.0, 6.1–6.9, 7.0–7.7 and ≥7.8 mmol/L), selected to include the FPG thresholds for impaired fasting glucose and diabetes. RPG was also modelled as a continuous variable to estimate risk associated with a 1 mmol/L higher level of RPG. The analysis for RPG was additionally adjusted for fasting time. For analyses involving more than two categories, all HRs are presented with ‘floating’ SEs to facilitate comparisons between groups.^12^

In sensitivity analyses, we examined the associations for diabetes and for RPG in subgroups defined by age-at-risk (35-59, 60-69, 70-79), sex, region, education, smoking, alcohol, physical activity, BMI, waist-to-hip ratio (WHR) and history of cardiovascular disease (CVD). We also repeated the analyses for diabetes excluding CRC cases that occurred in the first 2 or 5 years of follow-up. For the associations of diabetes with CRC risk, we further adjusted for diabetes medication (ie, metformin and insulin) and reported the HRs of CRC associated with diabetes medication. Statistical analyses were done using SAS V.9.3 and R V.2.14.2.

## Results

Among all 510 136 participants, the mean (SD) age was 51.5 (10.7) years and 59% were women. The prevalence of previously diagnosed and screen-detected diabetes was 3.1% and 2.7%, respectively. The mean (SD) RPG was 5.7 (1.1), 11.8 (5.7) and 13.3 (5.4) mmol/L among participants without any diabetes, with previously diagnosed diabetes and with screen-detected diabetes, respectively. Participants with diabetes were older and more likely to have higher levels of BMI, higher SBP, lower physical activity and to have CVD, hypertension and a family history of diabetes (table 1). Among all 16 000 participants with previously diagnosed diabetes, the median age at first diagnosis was 53 years and the median duration since diagnosis was 6.5 years. During approximately 10 years of follow-up, 3024 participants developed CRC between the ages of 40–79 years, including 1745 colon and 1716 rectal cancer cases.

**Table 1:** Baseline characteristics by diabetes status in CKB

Variable*			Diabetes status	
	No diabetes	Total	Previously diagnosed	Screen detected
	(n=480 130)	(n=30 006)	(n=16 000)	(n=14 006)
Age (SD), year	51.1 (10.6)	57.2 (9.6)	58.4 (9.1)	55.9 (9.9)
Female, %	59.0	61.7	62.3	61.0
Socioeconomic and lifestyle factors				
Urban resident, %	43.0	60.9	65.1	55.9
≥6 years of education, %	43.4	43.2	44.7	42.2
Household income≥35 000 RMB/year, %	24.7	24.3	24.5	24.1
Ever regular smoking, %				
Male	67.8	66.2	64.4	67.6
Female	2.8	3.1	3.1	3.0
Weekly drinking %				
Male	33.8	29.6	21.8	36.2
Female	2.1	1.3	0.7	1.9
Total physical activity (SD), MET-hours/day	21.3 (13.9)	18.8 (11.9)	17.5 (10.6)	19.8 (12.9)
Anthropometry and blood pressure				
Height (SD), cm	158.6 (8.3)	158.8 (8.4)	159.0 (8.3)	158.5 (8.4)
BMI (SD), kg/m^2^	23.6 (3.3)	24.9 (3.6)	24.6 (3.5)	25.1 (3.7)
Waist circumference (SD), cm	80.0 (9.6)	84.9 (10.0)	84.3 (9.8)	85.3 (10.3)
Hip circumference (SD), cm	90.9 (6.8)	92.2 (7.6)	91.8 (7.5)	92.6 (7.7)
Waist-to-hip ratio (SD)	0.88 (0.07)	0.92 (0.07)	0.92 (0.07)	0.92 (0.07)
Per cent body fat (SD)	27.9 (8.3)	30.4 (8.7)	29.4 (8.5)	31.1 (8.8)
BMI at age 25 (SD), kg/m^2^	21.9 (2.5)	22.8 (3.1)	23.2 (3.2)	22.5 (2.9)
SBP (SD), mm Hg	130.7 (21.0)	138.6 (22.6)	137.6 (22.6)	139.3 (22.7)
RPG (SD), mmol/L	5.7 (1.1)	12.6 (5.6)	11.8 (5.7)	13.3 (5.4)
Prior disease history, %				
CHD	2.8	5.2	6.9	3.2
Stroke or TIA	1.6	3.2	3.9	2.2
Hypertension	10.8	22.5	27.8	17.4
Family history of diabetes	4.5	12.2	16.1	9.2
Family history of cancer	13.9	14.1	15.1	13.3

*Results were adjusted for age, region and sex (where appropriate). 1 mmol/L=18 mg/dL.

BMI, body mass index; CHD, coronary heart disease; MET, metabolic equivalent of task; RPG, random plasma glucose; SBP, systolic blood pressure; TIA, transient ischaemic attack.

Diabetes was associated with an 18% higher risk of CRC (HR 1.18, 95% CI 1.04 to 1.33, table 2), with adjusted HRs of 1.19 (1.01 to 1.39) for colon cancer and 1.14 (0.96 to 1.35) for rectal cancer (p for heterogeneity 0.72). Additional adjustment for BMI attenuated the association for CRC (HR 1.14 (1.01 to 1.29), table 2). The associations for CRC persisted after excluding cases that occurred during the first 2 or 5 years of follow-up (online supplementary table 2) and were similar for incidence and mortality (p for heterogeneity 0.98, online supplementary table 3). When examining previously diagnosed diabetes and screen-detected diabetes separately, the HR was stronger for screen-detected diabetes (1.42 (1.21 to 1.67)), whereas the HR was non-significant for previously diagnosed diabetes (0.99 (0.84 to 1.18), online supplementary table 4).

**Table 2:** Adjusted HRs for colorectal cancer by diabetes status at baseline

	No. events	Rate, per 100 000	Model 1 HR (95% CI)	Model 2 HR (95% CI)
Colorectal cancer				
No diabetes	2732	569.0	Reference	Reference
Diabetes	292	973.1	1.18 (1.04 to 1.33)	1.14 (1.01 to 1.29)
Colon cancer				
No diabetes	1570	327.0	Reference	Reference
Diabetes	175	583.2	1.19 (1.01 to 1.39)	1.14 (0.97 to 1.34)
Rectal cancer				
No diabetes	1561	325.1	Reference	Reference
Diabetes	155	516.6	1.14 (0.96 to 1.35)	1.11 (0.94 to 1.32)

Model 1: stratified by age-at-risk, sex and region and adjusted for age at baseline, education, smoking, alcohol and total physical activity.

Model 2: Model 1 further adjusted for BMI.

BMI, body mass index.

When individuals with incident diabetes after baseline were also considered as exposed from their time of diagnosis (table 3), the associations were similar to diabetes status assessed at baseline for CRC (1.15 (0.95 to 1.38)), colon cancer (1.24 (0.98 to 1.56)) and rectal cancer (1.11 (0.86–1.43)). Likewise, the associations were similar for proximal and distal colon cancer and for distal and rectal cancer combined (online supplementary table 5). Compared with participants without diabetes (table 3), the risk of CRC was highest during 0 to <2 years following diabetes diagnosis (1.47 (1.10 to 2.28)), decreased with increasing duration of diabetes and became non-significant ≥10 years after diabetes diagnosis (0.95 (0.78 to 1.28)). Similar patterns were observed for colon and rectal cancer, although the decreasing trend appeared stronger for colon cancer (table 3).

**Table 3:** Adjusted HRs for colorectal cancer by duration of diabetes*

	Colorectal cancer		Colon cancer		Rectal cancer	
	No. events	HR (95% CI)	No. events	HR (95% CI)	No. events	HR (95% CI)
Incident diabetes	299	1.15 (0.96 to 1.38)	186	1.24 (0.98 to 1.56)	153	1.11 (0.86 to 1.43)
Diabetes						
No diabetes	2576	1.00 (0.96 to 1.07)	1466	1.00 (0.94 to 1.09)	1485	1.00 (0.94 to 1.09)
0 to <2 years	49	1.47 (1.10 to 2.28)	30	1.66 (1.16 to 2.85)	28	1.34 (0.89 to 2.49)
2 to <5 years	117	1.18 (0.94 to 1.67)	71	1.25 (0.93 to 1.94)	57	0.97 (0.69 to 1.63)
5 to <10 years	225	1.33 (1.13 to 1.69)	139	1.43 (1.17 to 1.94)	125	1.33 (1.07 to 1.85)
≥10 years	57	0.95 (0.78 to 1.28)	39	0.89 (0.68 to 1.33)	21	0.94 (0.72 to 1.42)
P for trend		0.03		0.02		0.36

The HR for incident diabetes was also adjusted for prevalent diabetes. P for trend was calculated among participants with diabetes.

For previously diagnosed diabetes and incident diabetes, duration was calculated as the time interval between age of diabetes diagnosis and age at risk.

For screen-detected diabetes, duration was calculated as the time interval between age at baseline and age at risk.

Duration of diabetes was missing in 20 participants.

*Estimates were stratified by age-at-risk, sex and region and adjusted for age at baseline, education, smoking, alcohol, total physical activity and BMI.

BMI, body mass index.

**Figure 1:**
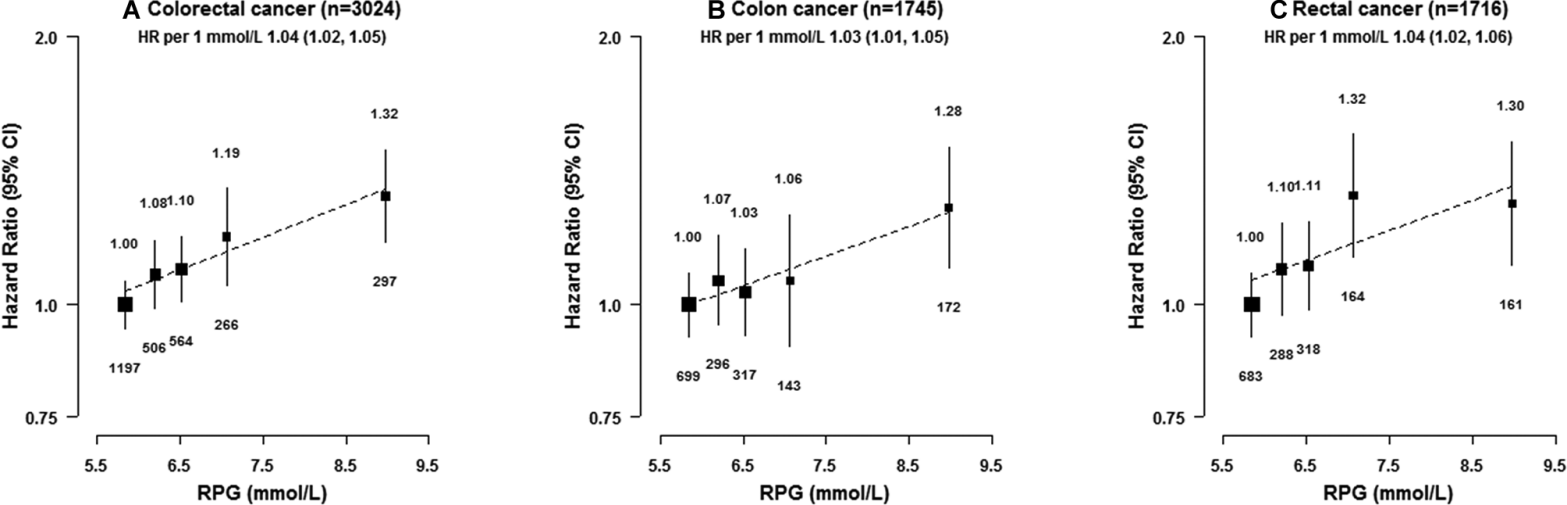
Adjusted HRs for colorectal cancer by levels of RPG among individuals without previously diagnosed diabetes at baseline. RPG levels for participants without previously diagnosed diabetes at baseline were classified as ≤5.5 (reference), 5.6–6.0, 6.1–6.9, 7.0–7.7 and ≥7.8 mmol/L. HRs were plotted against the mean usual RPG level in each group. Mean usual RPG levels were calculated as the mean RPG levels at the resurvey according to baseline RPG categories. The size of the boxes is proportional to the inverse of the variance of the log HRs. The models were stratified by age-at-risk, sex and region and adjusted for age at baseline, education, smoking, alcohol, total physical activity and fasting time. 1 mmol/L=18 mg/dL. RPG, random plasma glucose.

In participants without previously diagnosed diabetes, there was a positive association of RPG with risk of CRC (figure 1 and online supplementary table 6), with adjusted HRs of 1.00 (0.94 to 1.06) (reference), 1.11 (1.05 to 1.18), 1.10 (0.98 to 1.22) and 1.33 (1.18 to 1.49) for those with RPG of ≤5.5, 5.6–6.7, 6.8–7.7 and ≥7.8 mmol/L. Similar associations were observed for colon and rectal cancer (1.03 [1.01 to 1.05] vs 1.04 [1.02 to 1.06] per 1 mmol/L higher baseline RPG, *p* for heterogeneity 0.52, figure 1) and for CRC incidence and mortality (1.04 [1.02 to 1.05] vs 1.04 [1.01 to 1.07] per 1 mmol/L higher baseline RPG, *p* for heterogeneity 0.99, online supplementary table 3). As for diabetes, additional adjustment for BMI attenuated the association for CRC (HR 1.03 [1.02 to 1.05] per 1 mmol/L higher RPG, online supplementary table 6).

**Figure 2:**
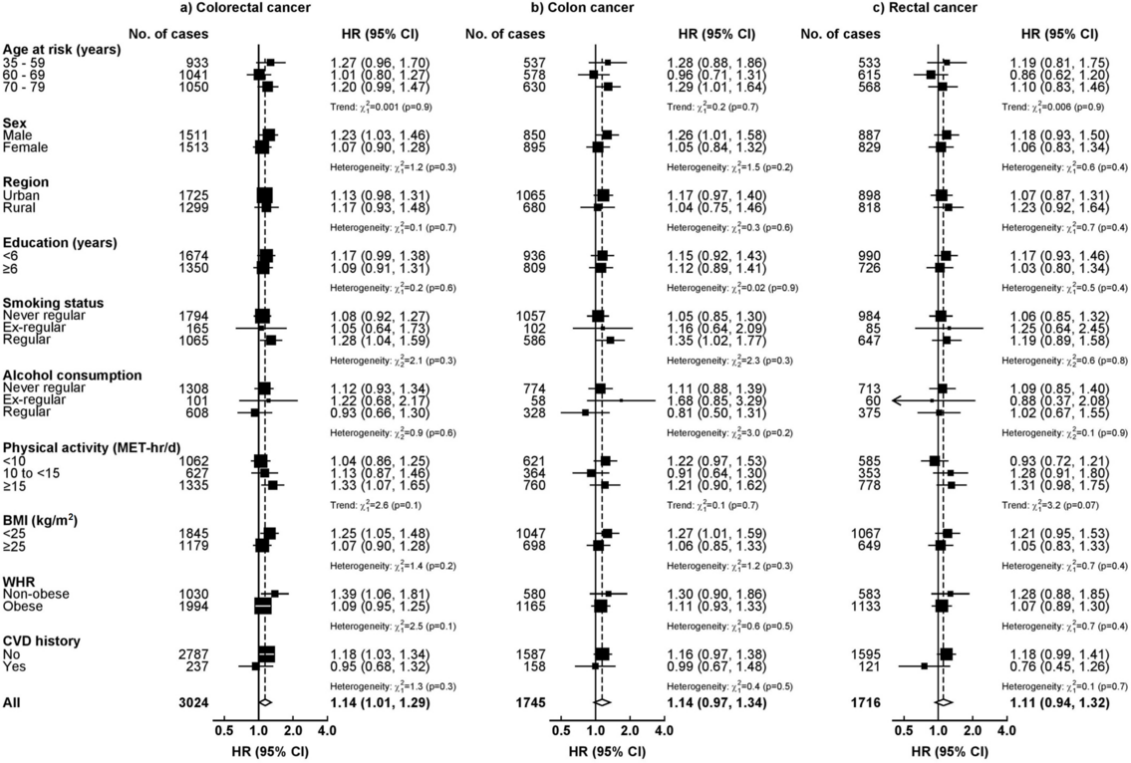
Adjusted HRs for colorectal cancer associated with diabetes in population subgroups. The models were stratified by age-at-risk, sex and region and adjusted for age at baseline, education, smoking, alcohol, total physical activity and BMI where appropriate. Boxes represent subgroup-specific estimates and diamonds represent the overall HR. The sizes of the boxes are proportional to the inverse of the variance of the log HRs. For WHR, obese participants included men with a WHR<0.85 and women with a WHR<0.90. BMI, body mass index; MET, metabolic equivalent of task; WHR, waist-to-hip ratio.

**Figure 3:**
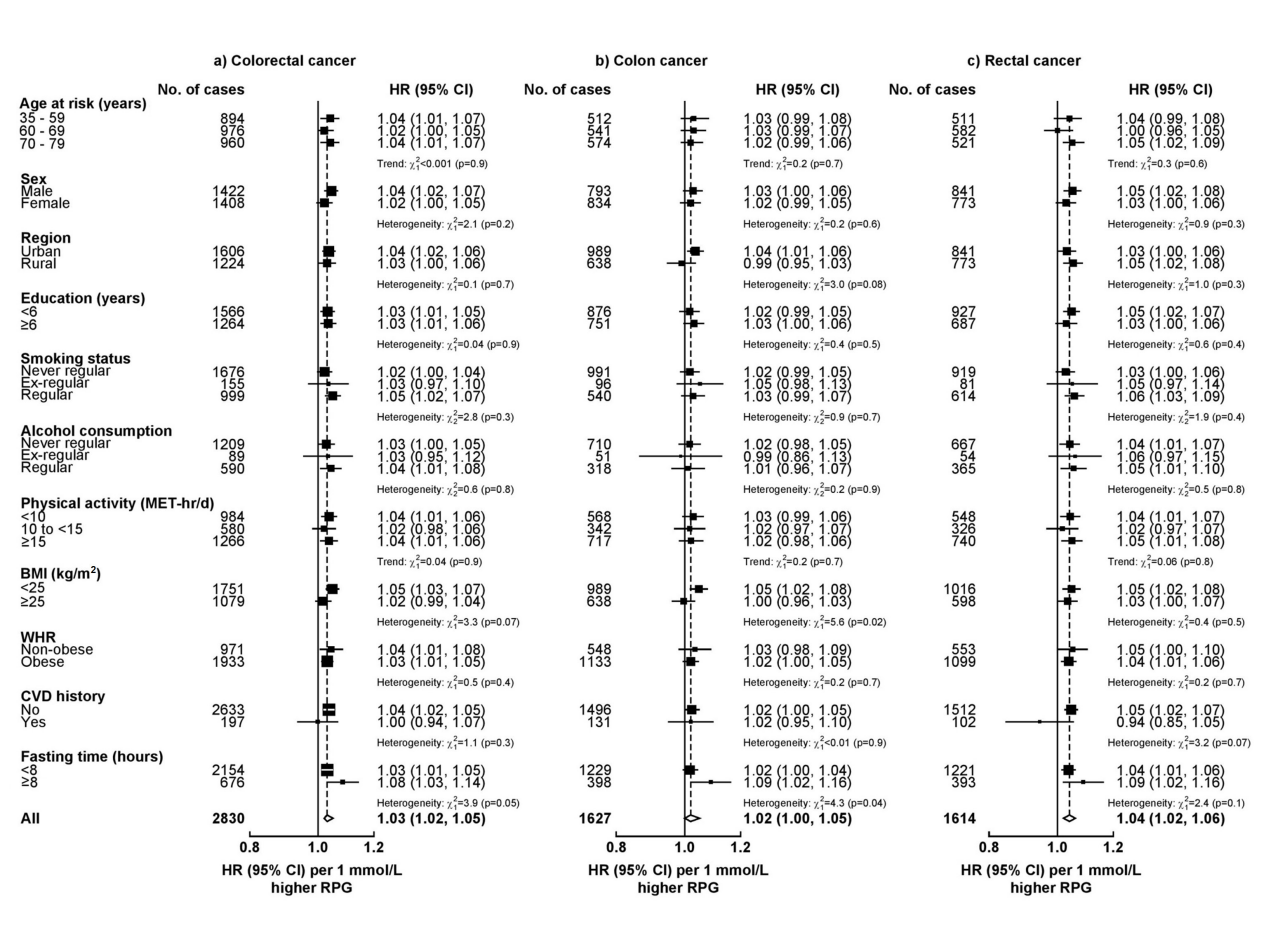
Adjusted HRs for colorectal cancer associated with 1 mmol/L higher RPG among individuals without previously diagnosed diabetes in population subgroups. Conventions as in figure 2. 1 mmol/L=18mg/dL. BMI, body mass index; MET, metabolic equivalent of task; RPG, random plasma glucose; WHR, waist-to-hip ratio.

In subgroup analyses, the overall patterns were similar comparing colon and rectal cancer. For both diabetes and RPG, the HRs for CRC appeared stronger in men than in women and in non-obese than obese participants, although heterogeneity tests were non-significant (figure 2 and figure 3). Likewise, the association of plasma glucose with risk of CRC was stronger for fasting than non-fasting participants (figure 3).

In sensitivity analyses, the associations of diabetes with risk of CRC attenuated when further adjusting for diabetes medication (online supplementary table 7). Compared with participants without diabetes, the HRs for CRC were similar among patients with diabetes on different medications (online supplementary table 8). The associations with risk of CRC were stronger for untreated diabetes than treated diabetes (online supplementary table 9).

## Discussion

In this Chinese population, we showed that individuals with diabetes had higher risk of CRC. The associations of diabetes with CRC appeared stronger in men than in women and were similar for colon and for rectal cancer. The risks of CRC appeared to decrease with increasing duration of diabetes and became non-significant 10 years after diabetes diagnosis. Among those without a prior diagnosis of diabetes, there was a positive association of RPG with CRC risk.

Previous prospective studies in Western and other East Asian populations have shown a positive association between diabetes and CRC risk. A meta-analysis of 29 prospective cohort studies (62 924 cases) reported a 27% higher risk of CRC associated with diabetes and showed similar associations by sex and by anatomical site.^4^ Although the magnitude of the risk estimate in the present study was somewhat lower than the meta-analysis, our estimate was comparable to two large prospective studies in Korea.^13 14^ These two studies also included both previously diagnosed diabetes and screen-detected diabetes defined by fasting blood glucose and had a comparable proportion of screen-detected diabetes as our study.^13 14^ Although previous prospective studies have shown inconsistent associations between duration of diabetes and CRC risk,^4^ two prospective cohort studies which used a time-dependent approach reported decreasing trends between duration of diabetes and CRC risk.^15 16^ A prospective cohort study in the USA reported a decreasing trend in CRC risk with longer duration of diabetes, with HRs of 2.36 (0.96 to 5.79), 1.71 (0.63 to 4.61) and 1.01 (0.48 to 3.12) for 4–8, 8–12 and ≥12 years of diabetes.^15^ Similarly, a record linkage study in Israel reported a decreasing trend between duration of diabetes and CRC risk, although the longest duration was 11 years.^16^ The high risk of CRC in the first few years following diagnosis of diabetes might be due to surveillance bias and reverse causality, while the relatively low risk might reflect hypoinsulinaemia and glycaemic control over the course of diabetes. In CKB, the decreasing trend between duration of diabetes and CRC risk might explain the null association between previously diagnosed diabetes and CRC risk in CKB.

In addition to diabetes, a few prospective cohort studies have reported on the association between blood glucose and CRC risk. A meta-analysis of six prospective cohort studies (62 814 CRC cases) reported a pooled RR of 1.015 (1.012 to 1.019) per 18 mg/dL higher FPG, with similar RRs by sex and by anatomical site.^5^ Our estimate for FPG was somewhat stronger than the meta-analysis (1.09 vs 1.015). Although in our study the magnitude of effect for RPG was weaker than FPG due to measurement errors (1.03 vs 1.09), we found a positive association between RPG and CRC risk. RPG may be a more practical and relevant measure than FPG as people spend most of the time in a non-fasting state. Furthermore, none of the previous studies excluded participants with diagnosed diabetes whose blood glucose levels might be controlled by medications. Our findings suggested that the positive association of blood glucose with risk of CRC continued down to below the diabetic and prediabetic ranges.

Hyperglycaemia and insulin resistance have been hypothesised as the underlying mechanisms linking diabetes and CRC.^17^ Insulin can directly promote growth and mitogen of cancer cells and can indirectly impact carcinogenesis by increasing bioavailability of insulin-like growth factor (IGF).^17 18^ Previous cohort studies have shown positive associations of fasting insulin and IGF-I with risk of CRC.^6 19^ On the other hand, over the course of diabetes, there might be a shift from initial hyperinsulinaemia to subsequent hypoinsulinaemia,^20^ potentially explaining the inverse trend between diabetes duration and CRC risk. Although a few prospective cohort studies have suggested that metformin use might lower risk of CRC among patients with diabetes, it has been suggested that these studies may have time-related bias (ie, immortal time bias and time-window bias).^21^ Indeed, randomised controlled trials showed that individuals with diabetes who used metformin had a similar risk of developing cancer compared with those who used other first-line therapies (eg, sulfonylureas).^22^ Despite the small number of cases among patients with diabetes, we found no evidence that diabetes medications were related to CRC risk (online supplementary table 6).

The strengths of the CKB include a prospective design, a large and diverse study population, large numbers of CRC cases by sex and by anatomical site and careful adjustment for other risk factors for CRC. One major limitation of CKB was that screen-detected diabetes was defined using RPG and therefore may be subject to misclassification. However, our result was consistent with previous studies that used fasting plasma glucose or postload plasma glucose to define screen-detected diabetes.

In summary, among Chinese adults, diabetes was associated with higher risk of CRC. Among participants without prior diagnosis of diabetes, RPG was positively associated with risk of CRC. The associations for diabetes and RPG did not seem to differ by sex or by anatomical site. Given that the majority of diabetes cases are undiagnosed in China, early detection of diabetes could help identify a group of individuals at higher risk of CRC. Although future studies are warranted, lifestyle modifications to prevent diabetes and to lower blood glucose levels may lower risk of developing CRC.

**What is already known on this subject**

- Studies conducted in Western populations have shown that diabetes is associated with higher risk of colorectal cancer (CRC).
- However, evidence has been inconclusive about the relevance of duration of diabetes and whether the associations differ by sex or by anatomical site.
- Moreover, evidence is limited whether blood glucose levels are associated with risk of CRC.

**What this study adds**

- In this Chinese population, we found that diabetes was associated with higher risk of CRC, with lower risk associated with longer duration of diabetes.
- Among participants without diabetes, there was a positive association of random plasma glucose (RPG) with CRC risk.
- The associations of diabetes and of RPG were similar in men and in women and for colon and for rectal cancer. Early detection of diabetes could help identify a group of individuals at higher risk of CRC.
