# Supplementary material for: Diabetes, plasma glucose and incidence of colorectal cancer in Chinese adults: a prospective study of 0.5 million people
Source: J Epidemiol Community Health. 2018 Jul 3;72(10):919–25. doi: 10.1136/jech-2018-210651 (PMC6161653; doi:10.1136/jech-2018-210651)
Supplement: Supplementary file 1 [file jech-2018-210651supp001.pdf]

Supplementary Material

Diabetes, plasma glucose and incidence of colorectal cancer in Chinese adults: a prospective study of 0.5 million people

Journal of Epidemiology and Community Health

Yuanjie Pang[1], ScM; Christiana Kartsonaki[1,2], DPhil; Yu Guo[3], MSc; Yiping Chen[1,2], DPhil; Ling Yang[1,2], PhD; Zheng Bian[3], MSc; Fiona Bragg[1], DPhil; Iona Y Millwood[1,2], DPhil; Leijia Shen[4], BSc; Songgen Zhou[5], BSc; Jiben Liu[6], BSc; Junshi Chen[7], MD; Liming Li[3], MD; Michael V Holmes[1,2,9], PhD; Zhengming Chen[1], DPhil

1. Clinical Trial Service Unit & Epidemiological Studies Unit (CTSU), Nuffield Department of Population Health, University of Oxford, Oxford, UK
2. Medical Research Council Population Health Research Unit (MRC PHRU), Nuffield Department of Population Health, University of Oxford, Oxford, UK
3. Chinese Academy of Medical Sciences, 9 Dongdan San Tiao, Beijing 100730, China
4. Tongxiang Renmin Hospital, Tongxiang 314511, China
5. Wuzhen Central Hospital, Tongxiang 314501, China
6. Yongqing Road Community Health Center, Qingdao 266041, China
7. National Center for Food Safety Risk Assessment, 37 Guangqu Road, Beijing 100021, China
8. School of Public Health, Peking University, Beijing 100191, China
9. National Institute for Health Research Oxford Biomedical Research Center, Oxford University Hospital, Old Road, Oxford OX3 7LE, UK

Address for correspondence

Christiana Kartsonaki
MRC PHRU, CTSU
Nuffield Department of Population Health
Old Road Campus
University of Oxford
Oxford, OX3 7LF, UK
Fax: 44-1865-743985
Email: christiana.kartsonaki@ndph.ox.ac.uk

Table of Contents

Supplementary Figure 1. Flow chart 3

Supplementary Table 1. Distribution and classification of colorectal and small intestine cancer by ICD-10 code 4

Supplementary Table 2. Adjusted HRs for colorectal cancer by diabetes status after excluding early years of follow-up 5

Supplementary Table 3. Adjusted HRs for colorectal cancer mortality by diabetes and levels of RPG among individuals without previously diagnosed diabetes 6

Supplementary Table 4. Adjusted HRs for colorectal cancer by diabetes status 7

Supplementary Table 5. Adjusted HRs for colon cancer per 1 SD higher in different measures of adiposity by anatomical subsite 8

Supplementary Table 6. Adjusted HRs for colorectal cancer by levels of RPG among participants without previously diagnosed diabetes 9

Supplementary Table 7. Adjusted HRs for colorectal cancer by diabetes status 10

Supplementary Table 8. Adjusted HRs for colorectal cancer by diabetes medications..... 11

Supplementary Table 9. Adjusted HRs for colorectal cancer by treated and untreated diabetes 12

Supplementary Figure 1. Flow chart

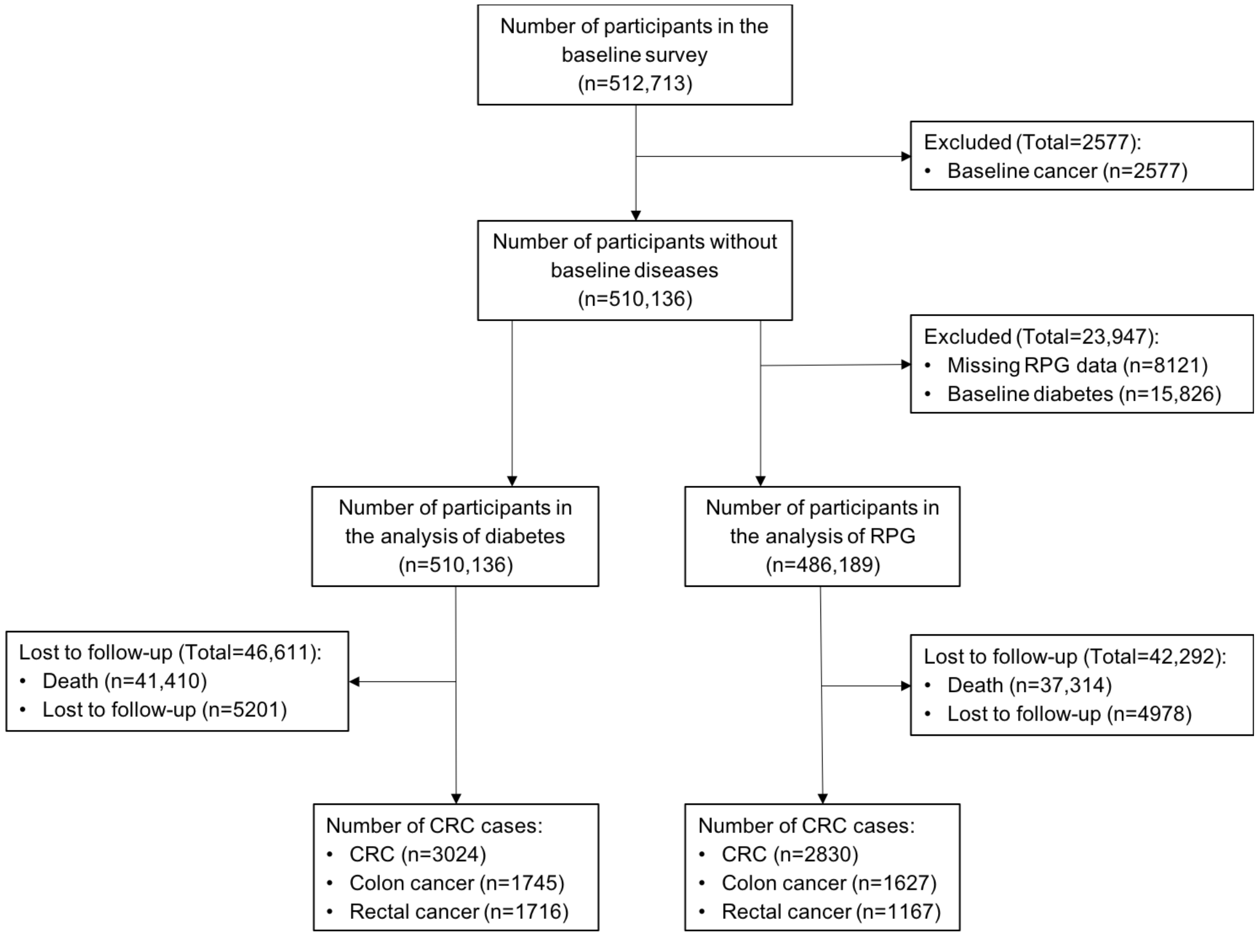

Supplementary Table 1. Distribution and classification of colorectal and small intestine cancer by ICD-10 code

ICD-10 code	Descriptions	No. of cases
C18.0	Caecum	63
C18.1	Appendix	15
C18.2	Ascending colon	135
C18.3	Hepatic flexure	37
C18.4	Transverse colon	45
C18.5	Splenic flexure	8
C18.6	Descending colon	34
C18.7	Sigmoid colon	276
C18.8	Overlapping lesion of colon	29
C18.9	Colon, unspecified	1474
**C18.0-5**	**Proximal colon**	**294**
**C18.6, C18.7**	**Distal colon**	**308**
**C18**	**Colon**	**1745**
**C19**	**Rectosigmoid junction**	**152**
**C20**	**Rectum**	**1716**
**C18-C20**	**Colorectum**	**3024**

Supplementary Table 2. Adjusted HRs for colorectal cancer by diabetes status after excluding early years of follow-up*

	No. events	Rate, Per 100,000	Excluding first 2 years of follow-up HR (95% CI)	No. events	Rate, Per 100,000	Excluding first 5 years of follow-up HR (95% CI)
**Colorectal cancer**						
No diabetes	2391	498.0	Reference	1632	340.0	Reference
Diabetes	252	839.8	1.13 (0.99, 1.30)	165	549.9	1.13 (0.96, 1.33)
**Colon cancer**						
No diabetes	1371	285.6	Reference	918	191.2	Reference
Diabetes	152	506.6	1.14 (0.96, 1.36)	93	309.9	1.10 (0.88, 1.36)
**Rectal cancer**						
No diabetes	1348	280.8	Reference	914	190.4	Reference
Diabetes	129	429.9	1.08 (0.90, 1.30)	93	309.9	1.19 (0.96, 1.49)

* Estimates were stratified by stratified by age-at-risk, sex, and region, and adjusted for age at baseline, education, smoking, alcohol, total physical activity, and BMI.

Supplementary Table 3. Adjusted HRs for colorectal cancer mortality by diabetes and levels of RPG among individuals without previously diagnosed diabetes

	No. events	Rate, Per 100,000	Model 1 HR (95% CI)	Model 2 HR (95% CI)
**Colorectal cancer**				
Diabetes				
No	866	180.4	Reference	Reference
Yes	95	316.6	1.18 (0.95, 1.47)	1.17 (0.94, 1.45)
RPG				
≤ 5.5	373	153.7	1.00 (0.90, 1.12)	1.00 (0.90, 1.12)
5.6 - 6.7	317	200.3	1.10 (0.99, 1.23)	1.10 (0.99, 1.22)
6.8 - 7.7	99	193.9	0.97 (0.80, 1.19)	0.97 (0.79, 1.18)
≥ 7.8	108	316.9	1.48 (1.22, 1.80)	1.47 (1.21, 1.79)
Effects per 1 mmol/L	***897***	***185.6***	1.04 (1.01, 1.07)	1.04 (1.01, 1.07)
p for trend			*<0.001*	*0.01*
**Colon cancer**				
Diabetes				
No	379	78.9	Reference	Reference
Yes	50	166.6	1.29 (0.95, 1.74)	1.27 (0.94, 1.71)
RPG				
≤ 5.5	147	60.6	1.00 (0.84, 1.19)	1.00 (0.84, 1.19)
5.6 - 6.7	147	92.9	1.30 (1.11, 1.52)	1.29 (1.10, 1.51)
6.8 - 7.7	44	86.2	1.11 (0.82, 1.50)	1.10 (0.82, 1.49)
≥ 7.8	60	176.1	2.20 (1.69, 2.85)	2.17 (1.67, 2.82)
Effects per 1 mmol/L	***398***	***81.9***	1.07 (1.04, 1.11)	1.07 (1.04, 1.11)
p for trend			*<0.001*	*<0.001*
**Rectal cancer**				
Diabetes				
No	475	98.9	Reference	Reference
Yes	43	143.3	1.07 (0.78, 1.47)	1.06 (0.77, 1.46)
RPG				
≤ 5.5	222	91.5	1.00 (0.87, 1.15)	1.00 (0.87, 1.15)
5.6 - 6.7	163	103.0	0.96 (0.82, 1.11)	0.96 (0.82, 1.11)
6.8 - 7.7	54	105.8	0.89 (0.68, 1.17)	0.89 (0.68, 1.16)
≥ 7.8	46	135.0	1.03 (0.77, 1.38)	1.02 (0.76, 1.37)
Effects per 1 mmol/L	***485***	***99.8***	1.00 (0.96, 1.05)	1.00 (0.95, 1.05)
p for trend			*0.98*	*0.96*

Model 1: stratified by age-at-risk, sex, and region, and adjusted for age at baseline, education, smoking, alcohol, total physical activity, and fasting time (for RPG).
Model 2: Model 1 plus BMI.

Supplementary Table 4. Adjusted HRs for colorectal cancer by diabetes status

	No. events	Rate, Per 100,000	Model 1 HR (95% CI)	Model 2 HR (95% CI)
**Colorectal cancer**				
No diabetes	2732	569.1	1.00 (0.96, 1.04)	1.00 (0.96, 1.05)
Previously diagnosed diabetes	144	900.0	0.99 (0.84, 1.18)	0.98 (0.83, 1.16)
Screen-detected diabetes	148	1056.7	1.42 (1.21, 1.67)	1.37 (1.17, 1.61)
**Colon cancer**				
No diabetes	1570	327.0	1.00 (0.95, 1.06)	1.00 (0.94, 1.06)
Previously diagnosed diabetes	90	562.5	1.03 (0.84, 1.27)	1.00 (0.81, 1.24)
Screen-detected diabetes	85	606.9	1.39 (1.13, 1.72)	1.33 (1.08, 1.65)
**Rectal cancer**				
No diabetes	1561	325.2	1.00 (0.94, 1.06)	1.00 (0.94, 1.06)
Previously diagnosed diabetes	70	437.5	0.90 (0.71, 1.14)	0.88 (0.70, 1.12)
Screen-detected diabetes	85	606.9	1.46 (1.18, 1.81)	1.42 (1.15, 1.76)

Model 1: stratified by age-at-risk, sex, and region, and adjusted for age at baseline, education, smoking, alcohol, and total physical activity.
Model 2: Model 1 plus BMI.

Supplementary Table 5. Adjusted HRs for colon cancer per 1 SD higher in different measures of adiposity by anatomical subsite*

	Total colon<br>HR (95% CI)	Proximal colon<br>HR (95% CI)	Distal colon<br>HR (95% CI)	Distal colon + rectal<br>HR (95% CI)
No. of cases	1745	294	308	2044
Diabetes	1.14 (0.83, 1.56)	0.64 (0.20, 2.09)	0.66 (0.20, 2.15)	1.02 (0.74, 1.40)
RPG per 1 mmol/L	1.03 (0.99, 1.08)	1.01 (0.87, 1.16)	0.99 (0.85, 1.15)	1.03 (0.99, 1.08)

ICD-10: total colon, C18; proximal colon, C18.0-5; distal colon, C18.6, C18.7; distal colon + rectal, C18.6, C18.7, C19, C20.
* Model was stratified by age-at-risk, sex, and region, and adjusted for age at baseline, education, smoking, alcohol, total physical activity, and fasting time (for RPG).

Supplementary Table 6. Adjusted HRs for colorectal cancer by levels of RPG among participants without previously diagnosed diabetes

	No. events	Rate, Per 100,000	Model 1 HR (95% CI)	Model 2 HR (95% CI)
**Colorectal cancer**				
≤ 5.5	1197	493.4	1.00 (0.94, 1.06)	1.00 (0.94, 1.06)
5.6 - 6.7	998	630.7	1.11 (1.05, 1.18)	1.10 (1.04, 1.17)
6.8 - 7.7	338	663.9	1.10 (0.98, 1.22)	1.08 (0.97, 1.20)
≥ 7.8	297	871.6	1.33 (1.18, 1.49)	1.29 (1.15, 1.45)
Effects per 1 mmol/L	***2830***	***582.2***	1.04 (1.02, 1.05)	1.03 (1.02, 1.05)
p for trend			*<0.001*	*<0.001*
**Colon cancer**				
≤ 5.5	699	288.1	1.00 (0.92, 1.08)	1.00 (0.92, 1.08)
5.6 - 6.7	575	363.4	1.08 (0.99, 1.17)	1.06 (0.98, 1.15)
6.8 - 7.7	181	355.5	0.98 (0.84, 1.13)	0.95 (0.82, 1.10)
≥ 7.8	172	504.8	1.29 (1.11, 1.50)	1.24 (1.06, 1.44)
Effects per 1 mmol/L	***1627***	***334.7***	1.03 (1.01, 1.05)	1.02 (1.00, 1.05)
p for trend			*0.01*	*0.04*
**Rectal cancer**				
≤ 5.5	683	281.5	1.00 (0.92, 1.08)	1.00 (0.92, 1.08)
5.6 - 6.7	562	355.2	1.12 (1.03, 1.21)	1.11 (1.03, 1.20)
6.8 - 7.7	208	408.6	1.22 (1.06, 1.40)	1.20 (1.05, 1.38)
≥ 7.8	161	472.5	1.30 (1.11, 1.52)	1.27 (1.09, 1.49)
Effects per 1 mmol/L	***1614***	***332.0***	1.04 (1.02, 1.06)	1.04 (1.02, 1.06)
p for trend			*<0.001*	*<0.001*

Model 1: stratified by age-at-risk, sex, and region, and adjusted for age at baseline, education, smoking, alcohol, total physical activity, and fasting time.
Model 2: Model 1 plus BMI.

Supplementary Table 7. Adjusted HRs for colorectal cancer by diabetes status

	No. events	Model 1 HR (95% CI)	Model 2 HR (95% CI)
**Colorectal cancer**			
No diabetes	2732	Reference	Reference
Diabetes	292	1.18 (1.04, 1.33)	0.85 (0.64, 1.11)
**Colon cancer**			
No diabetes	1570	Reference	Reference
Diabetes	175	1.19 (1.01, 1.39)	0.96 (0.68, 1.35)
**Rectal cancer**			
No diabetes	1561	Reference	Reference
Diabetes	155	1.14 (0.96, 1.35)	0.67 (0.45, 1.00)

Model 1: stratified by age-at-risk, sex, and region, and adjusted for age at baseline, education, smoking, alcohol, and total physical activity.
Model 2: Model 1 plus diabetes medication.

Supplementary Table 8. Adjusted HRs for colorectal cancer by diabetes medications*

	No. events	HR (95% CI)
**Colorectal cancer**		
No diabetes	2732	1.00 (0.95, 1.05)
No medication	31	1.03 (0.73, 1.47)
Metformin	71	0.94 (0.75, 1.19)
Insulin	19	1.29 (0.82, 2.02)
Metformin and insulin	1	0.31 (0.04, 2.23)
**Colon cancer**		
No diabetes	1570	1.00 (0.94, 1.07)
No medication	19	1.04 (0.66, 1.63)
Metformin	46	1.03 (0.77, 1.38)
Insulin	15	1.65 (0.99, 2.75)
Metformin and insulin	0	--
**Rectal cancer**		
No diabetes	1561	1.00 (0.94, 1.07)
No medication	13	0.81 (0.47, 1.40)
Metformin	36	0.87 (0.63, 1.21)
Insulin	7	0.95 (0.45, 1.99)
Metformin and insulin	1	0.58 (0.08, 4.11)

* Estimates were stratified by age-at-risk, sex, and region, and adjusted for age at baseline, education, smoking, alcohol, and total physical activity.

Supplementary Table 9. Adjusted HRs for colorectal cancer by treated and untreated diabetes*

	No. events	Rate, Per 100,000	Model 1 HR (95% CI)	Model 2 HR (95% CI)
**Colorectal cancer**				
No diabetes	2732	569.1	1.00 (0.96, 1.05)	1.00 (0.96, 1.05)
Treated diabetes	91	568.8	0.98 (0.80, 1.20)	0.95 (0.78, 1.17)
Untreated diabetes	179	1278.0	1.34 (1.15, 1.55)	1.29 (1.12, 1.49)
**Colon cancer**				
No diabetes	1570	327.0	1.00 (0.94, 1.06)	1.00 (0.94, 1.06)
Treated diabetes	61	381.3	1.10 (0.85, 1.41)	1.06 (0.83, 1.37)
Untreated diabetes	104	742.6	1.31 (1.08, 1.59)	1.26 (1.04, 1.52)
**Rectal cancer**				
No diabetes	1561	325.2	1.00 (0.94, 1.06)	1.00 (0.94, 1.06)
Treated diabetes	44	275.0	0.88 (0.65, 1.18)	0.86 (0.64, 1.16)
Untreated diabetes	98	699.7	1.32 (1.09, 1.61)	1.29 (1.06, 1.57)

* Treated diabetes includes previously diagnosed diabetes on medication, while untreated includes screen-detected and previous diagnosed not on medication.
Model 1: stratified by age-at-risk, sex, and region, and adjusted for age at baseline, education, smoking, alcohol, and total physical activity.
Model 2: Model 1 plus BMI.
